# Astrocyte derived TSP2 contributes to synaptic alteration and visual dysfunction in retinal ischemia/reperfusion injury

**DOI:** 10.1186/s13578-022-00932-1

**Published:** 2022-12-05

**Authors:** Tu Hu, Shuhan Meng, Qianyue Zhang, Shuang Song, Cheng Tan, Jufang Huang, Dan Chen

**Affiliations:** 1 Department of Anatomy and Neurobiology, School of Basic Medical Science, Central South University, No. 172 Tongzipo Road, Changsha, 410013 Hunan People’s Republic of China; 2 Eye Center of Xiangya Hospital, Central South University, No. 87 Xiangya Road, Changsha, 410008 Hunan People’s Republic of China; 3 Hunan Key Laboratory of Ophthalmology, Changsha, 410008 Hunan People’s Republic of China; 4 National Clinical Research Center for Geriatric Disorders, Xiangya Hospital, Central South University, Changsha, 410008 Hunan People’s Republic of China; 5 XiangYa School of Public Health, Central South University, No.238 Xiangya Road, Changsha, 410078 Hunan People’s Republic of China; 6 Xiangya School of Medicine, Central South University, No. 172 Tongzipo Road, Changsha, 410013 Hunan People’s Republic of China

**Keywords:** Retinal ischemia/reperfusion, Axons, Compensatory synaptic remodeling, Visual dysfunction, Astrocyte, Thrombospondin 2, α2δ1

## Abstract

**Background:**

Despite current intervention measures/therapies are able to ameliorate neuronal death following retinal injuries/diseases, the recovery of visual function remains unsatisfactory. Previous studies revealed that the retinal synapse and neurite changed during the early stage after retinopathy, which was considered to be detrimental to visual signal transmission. However, the specific profiles and the mechanisms underlying retinal neurite and synaptic alteration after retinal pathologies remain poorly understood.

**Methods:**

Here, we revealed the spatiotemporal pattern of neurite and synaptic alteration following retinal pathologies using a rat model of acute RI/R induced by high intraocular pressure (HIOP) with Western blotting, Immunofluorescence, and electron microscopy. We further explored the potential role of activated astrocytes and their derived thrombospondin 2 (TSP2) in RI/R induced retinal neurite and synaptic alteration and visual dysfunction through viral transduction and drug injection.

**Results:**

We found a defasciculation of RGC axons, a compensatory increase of presynaptic proteins (synaptophysin and synapsin 1) and synaptic vesicles between bipolar cells and ganglion cells in the inner plexiform layer (IPL), and the degenerated visual function preceded the neuronal death in rat retinae. These events were accompanied by the activation of astrocytes. Furthermore, we showed that suppressing the activation of astrocytes (intravitreal injection of fluorocitric acid, FC), TSP2 knockdown (TSP2 shRNA-AAV transduction), and competitively inhibiting the binding of TSP2 and α2δ1 (intraperitoneal injection of gabapentin, GBP) effectively alleviated the retinal synaptic and neurite alteration and the visual dysfunction following RI/R injury.

**Conclusions:**

(1) At the early stage following RI/R injury, the rat retinae develop a degeneration of ganglion cell axons and the resulting compensatory synaptic remodeling between bipolar cells and ganglion cells in IPL. These changes occur earlier than the massive loss of neurons in the ganglion cell layer (GCL). (2) Activated astrocytes may secret TSP2, which bind to α2δ1, to mediate the degeneration of rat retinal ganglion cell axons, compensatory synaptic remodeling in IPL, and visual dysfunction following RI/R injury.

## Introduction

Retinal ischemia/reperfusion (RI/R), due to the absence of effective therapeutic measures, remains a leading cause of visual impairment and blindness [1]. This cause of visual impairment and blindness is becoming increasingly prevalent, especially in the aging population [2, 3]. When hypoxia–ischemia occurs, there is a multi-faceted cascade of events that starts out in retinal neurons, including the rapid depletion of ATP, the change to anaerobic metabolism, the accumulation of lactic acid, and failure of the ATP-dependent Na^+^/K^+^ pump. During the process of reperfusion, the recovery of blood supply and oxidative metabolism triggers an increase of radical oxygen species (ROS), high intracellular Ca^2+^, and up-regulation of pro-inflammatory genes [4]. All these pathological processes result in cellular edema and eventual neuronal death. Thus, a substantial effort is made to elucidate the complex mechanisms for neuronal death in retinae following retinal I/R injury. Hypotensive drugs (ß-blockers, α-agonists, and prostaglandins), Ca^2+^ channel blockers, NMDA antagonists and nitric oxide synthase inhibitors have been used as neuroprotective drugs against neuronal death. However, despite the therapeutically-induced retinal neurons increase, the recovery of visual function remains unsatisfactory [5, 6].

Besides the neuronal survive, the homeostasis of synapses and neurites is also vital for the function of visual processing [7]. Previous studies demonstrated that neurons apoptosis and neurite alternation are pivotal features in retinal injuries, and both led to vision dysfunction albeit separately [8, 9]. Accumulating evidences has shown the alteration of synapses and neurites preceded the neuronal apoptosis following retinal injuries or diseases [8, 10]. In chronic intraocular pressure, Park et al. found that this leads to an increase of synaptic vesicle protein and immature synapse formation between retinal ganglion cells (RGCs) and bipolar cells [11]. Robert K P Sullivan et al. illustrated that aged human retinal neurons also have the capacity to form new synapses in the human age-related macular degeneration retina [12]. Our previous data also showed that the presynaptic protein synaptophysin (SYN) increased in inner plexiform layers (IPLs) early after elevated IOP [13]. These events will surely affect the transmission of visual signals. Thus, the synaptic and neurite changes should be considered as the target of intervention measures/therapies for retinal injuries or diseases. However, the detailed pattern and the key regulating factors underlying the synaptic and neurite changes under retinal pathological conditions are far from clear.

During the development of the central nervous system (CNS), astrocytes put the initial synaptogenesis in the right place at the right time [14]. Neurons respond to the astrocyte secreted signals (Thrombospondins, Glypicans [15], Sparc II [16] and so on) to form and prune the mature synapses. Astrocytes also regulate multiple aspects of synaptic plasticity and function from development through to adulthood [17]. Meanwhile, the brain/peripheral nerve injury-induced synaptogenesis and synaptic plasticity retain an important role of astrocytes [18, 19]. Our group previously found that retinal astrocyte activation and the increased astrocyte-derived thrombospondin 2 (TSP2) occurred early following elevated hydrostatic pressure in vitro, which was temporally consistent with the increased presynaptic components [20]. These implied that the activated astrocyte might be associated with the synaptic and neurite changes following RI/R. Furthermore, TSP2 could be present as a signal to control the formation of structural glutamatergic synapses via α2δ1 (a subunit of voltage-gated calcium channel). All of the synaptic structural elements could respond to the astrocyte-derived “TSP2-α2δ1” signal, including presynaptic vesicles, active release sites and postsynaptic density [15].

In this article, we used a rat model of acute RI/R induced by high intraocular pressure (HIOP) to explore the role of astrocyte-derived TSP2 in the retinal synaptic and neurite alteration and visual dysfunction following RI/R. We tried to identify new evidences for retinal repair strategies and better visual function recovery including optimal intervention time and new targets.

## Materials and methods

### Animals and grouping

Ten-week-old female Sprague–Dawley (SD) rats (200–220 g), specific pathogen free (SPF), available from the animal center of Central South University, were used for experiments in the present study. All animals were housed in cages at the Central South University Animal Department (Changsha, China) under controlled temperature (21 ± 1 °C), humidity (55 ± 5%), and a 12 h light/dark circle. Food and water were available ad libitum. The physical condition of rats was monitored each day during the experiment. The rats with cataracts, or ocular fundus hemorrhage or infection were excluded.

All rats were randomly allocated into seven groups: (i) Control group; (ii) I/R group, rats were treated with anterior chamber compression; (iii) I/R + fluorinated citric acid (FC) group, rats were intravitreally injected with 2 μL FC after the induction of RI/R; (iv) I/R + TSP2 shRNA group, rats were intravitreally injected of 2 μL of TSP2 shRNA adeno-associated virus (AAV) liquid 28 d before RI/R treatment; (v) I/R + scramble RNA group, rats were intravitreally injected of 2 μL of scramble RNA AAV liquid 28 d before RI/R treatment; (vi) I/R + gabapentin (GBP) group, rats were intraperitoneally injected with GBP (100 mg/kg) before and 5 h after RI/R treatment; (vii) I/R + PB group, rats were intravitreally/intraperitoneally injected the same dose of 0.01 mol/L PB.

All animals were treated according to the Association for Research in Vision and Ophthalmology Resolution on the Use of Animals in Research. All animal experiments were reviewed and approved by the Medical Ethics Committee at Xiangya Hospital of Central South University (Approval ID: 2019030519).

### RI/R and administration of FC/GBP

All rats in RI/R groups were treated as described previously [21]. Briefly, a 30-gauge infusion needle connected to the installation instrument with sterile saline was inserted into the anterior chambers of the eyes of the animals after anesthetization (a 1:1 mixture solution (0.4 mL/100 g) of 25% Urethane and 10% chloral hydrate). The intraocular pressure was slowly elevated to 14.63 kPa (110 mmHg), maintained for 60 min, and then gradually lowered to normal pressure. The administration of FC was performed as described previously [22]: After the induction of RI/R, rats were intravitreally injected with 2 μL FC (Sigma Aldrich, USA, 16 nmol/L) or vehicle (0.01 mol/L PB). The needle of the micro-syringe was kept in the vitreous chamber for 2 min and then slowly removed. The administration of GBP was referred to the procedures as a previous description: rats were intraperitoneally injected with GBP (100 mg/kg) or the same volume of vehicle (0.01 mol/L PB) before and 5 h after RI/R.

### TSP2 shRNA-AAV transduction

TSP2 shRNA-AAV, Scramble RNA-AAV and AAV-GFP were obtained from HANBIO (Shang Hai, China) as follows: Gene ID: NM 001,169,138.1, the length of CDS: 3519 bp, sources: Rat, interfere sequence: 5′GCAGAUAUCUGCUUCUAA dTdT3′, serotype: II, titer: 1 × 10^12^. Intravitreal injection was treated as described previously [23], Briefly, the rats were anesthetized with a 1:1 mixture solution (0.4 mL/100 g) of 25% Urethane and 10% chloral hydrate and placed on a heating pad that maintained their body temperature at 35–36 °C throughout the experiments. After anesthesia and pupil dilation with 1% atropine, intravitreal injection of 2 μL of TSP2 shRNA AAV liquid/Scramble RNA AAV liquid/ AAV-GFP liquid was made into one eye of each rat 1 mm behind the limbus, using a 33-gauge needle (Hamilton, Reno, NV, USA) under a surgical microscope. After injection, the rats were maintained normally for 28 days to allow sufficient retinal transduction before the subsequent experiments.

### Tissue preparation

Rats were trans-cardiac perfused with saline and then 4% paraformaldehyde in 0.1 M phosphate buffer (PB, pH 7.4) after being deeply anesthetized. For the morphological assay, eyeballs were enucleated, and the cornea, lens and vitreous body were removed. Some remaining eye cups were postfixed in 4% PF overnight, immersed (15% to 30% sucrose solutions) at 4 °C for cryoprotection, then were prepared into 14 μm thick cross-sections successively on slices. Some eye cups were postfixed in 4% PF for 1 h, retinae were dissected, cut into four petals in 24 well plates. For western blotting, retinae were dissected from deeply anesthetized rats and then weighed and quickly frozen on dry ice and stored at − 80 °C for further homogenization.

### Immunofluorescence/Double Immunofluorescence

For immunofluorescence of phosphorylated neurofilament (pNF), retinal mounts were pre-incubated for 2 h in 5% donkey serum (Sigma, USA) at room temperature (RT), and then incubated with the anti-pNF antibody (1:200, Mo, BioLegend, USA, Catalog: 801602)/anti-GFAP antibody (1:1000, Mo, Sigma Aldrich, USA) at 4 °C overnight. After several rinses with phosphate Buffered Saline (PBS), mounts were reacted with 488-conjugated donkey anti-mouse secondary antibody (1:400, Jackson ImmunoResearch, USA). The sections were finally covered with a mounting medium containing 40,6-diamidino-2-phenylindole (DAPI, VEC- TOR, CA, USA). For immunofluorescence of GFAP and NeuN, retinal sections were pre-incubated for 1 h in 5% donkey serum (Sigma, USA) at room temperature (RT), and then incubated with anti-GFAP (1:1000, Mo, Sigma Aldrich, USA)/anti-NeuN (1:1000, Mo, Abcam, UK, ab104224) at 4 °C overnight. After several rinses with phosphate Buffered Saline (PBS), mounts were reacted with 488-conjugated donkey anti-mouse secondary antibody (1:400, Jackson ImmunoResearch, USA). The sections were finally covered with a mounting medium containing 40,6-diamidino-2-phenylindole (DAPI, VEC- TOR, CA, USA).

For double immunofluorescence of SYN and PKC-α/GFAP and TSP2, retinal sections were pre-incubated for 1 h in 5% donkey serum (Sigma, USA) at room temperature (RT), and then incubated with anti-SYN (1:500, Rb, Abcam, UK, ab32127) and anti-PKC-α (1:200, Mo, Santa Cruz, USA, sc-8393)/anti-GFAP (1:1000, Mo, Sigma Aldrich, USA) and anti-TSP2 (1:1000, Rb, Abcam, ab84469, Cambridge, UK) at 4 °C overnight. After several rinses with phosphate Buffered Saline (PBS), mounts were reacted with 488-conjugated donkey anti-rabbit secondary antibody (1:400, Jackson ImmunoResearch, USA) and 594-conjugated donkey anti-mouse secondary antibody (1:400, Jackson ImmunoResearch, USA). The sections were finally covered with a mounting medium containing 40,6-diamidino-2-phenylindole (DAPI, VECTOR, CA, USA).

### Transmission electron microscopy

Electron microscopy was conducted using retinal electron microscopic sections from controls and post-surgery (three rats per group). Retina tissues were cut into 1 mm^3^ cube with a vibratome and rinsed with clean saline. Tissues were fixed in 2.5% glutarol solution for 1 h at room temperature or 3 h at 4 °C and then with 1% osmium tetroxide in 0.1 mmol/L cacodylate buffer for 2 h. After rinsing with DDW, sections were treated with 1% aqueous uranyl acetate overnight, dehydrated in ethanol solutions of increasing concentration, up to 100%, followed by dry acetone, and then embedded in durcupan ACM. Ultrathin Sects. (0.1 μm) were cut and mounted on Formvar-coated slot grids, stained with 3% lead citrate, and examined with a HT7700 transmission electron microscope (Hitachi, Tokyo, Japan).

### Western Blotting

As previously detailed [21], retinae were homogenized by sonication on ice in a digestion buffer containing a cocktail of protease inhibitors (Sigma, MO, USA). Sonication-digested homogenates were treated with centrifugation, protein concentration determination and degeneration, respectively. Polypeptides were loaded in each lane of 4–20% linear gradient Tris–HCl ready gel (Bio-Rad, CA, USA) and the gel was run in electrophoresis at the voltage of 100 mV. And then, the polypeptides in the gel were electrotransferred to Trans-Blot pure nitrocellulose membrane (Bio-Rad, CA, USA). Following this, they were blocked by 5% non-fat milk for 1 h, the membranes were incubated with anti-GFAP(1:1000, Mo, Sigma Aldrich, USA)/anti-TSP2(1:1000, Rb, Abcam, UK)/anti-α2δ1(1:400, Mo, Abcam, UK)/anti-SYN(1:2000, Rb, Abcam, UK, ab32127)/anti-synapsin 1(1:1000, Rb, Abcam, UK, ab254349)/hormer 1b/1c (1:1000, Mo, Santa Cruz, USA, sc-55463)/ anti-mouse GAPDH (1:2000, Mo, Beyotime, China, AF0006) antibodies overnight and then placed in HRP-conjugated secondary antibodies (1:20,000, Bio-Rad, CA, USA) for 2 h to develop.

### qRT–PCR

TSP2 mRNA expression was confirmed using quantitative real-time PCR (qRT–PCR). The total RNA was reverse-transcribed to cDNA using GoScriptTM Reverse Transcription System (A5001, Promega Corporation, CA, USA) according to the manufacturer’s protocol. qRT–PCR was performed using a real-time fluorescence quantitative PCR instrument (ABI 7500, USA). An amplification mixture was prepared using Hieff^®^ qPCR SYBR Green Master Mix (11202ES03, Yeasen Corporation, China) according to the manufacturer's protocol, which contained 10.0 μL SYBR Green Master Mix, 0.4 μL forward primer, 0.4 μL reverse primer, 2 μL cDNA, and 20 μL ddH_2_O. The results were normalized to GAPDH expression. The primers (synthesized by Tsingke Biological Technology Co., Ltd., Beijing, China) are listed as follows: The primers used were as follows: *Tsp2*: forward, GTAGGTTTTGACGAGTTTGG, reverse, TCCACATCACCACATAGAAG; *GAPDH*: forward, CGTCCCGTAGACAAAATGGTGAA, reverse, GCCGTGAGTGGAGTCATACTGGAACA. The relative expression levels of mRNAs were depicted as 2^−△△Ct^.

### f-VEP

The rats were anesthetized with a 1:1 mixture solution (0.4 mL/100 g) of 25% Urethane and 10% chloral hydrate and placed on a heating pad that maintained their body temperature at 35–36 °C throughout the experiments. pupil dilation with 1% atropine. The reference electrode is fixed at the buccal masseter muscle on the same side as the test eyeball, and the recording electrode is fixed at 1–2 mm of the coronal suture of the skull and 2–3 mm of the sagittal suture. An earth clip, serving as a ground electrode, was placed along the tail. The test eye was exposed, while the opposite eyeball was shielded by an opaque eye shield. The visual stimulus was provided by Espion 2 visual electrophysiometer exposure device from Diagnosys (USA), backlight: 0 cd × s/m^2^ with white light: 600 cd/m^2^ × 5 ms, 1 Hz. Each f-VEP report was obtained from the average value of 64 visual stimuli, the VEP signal was expanded, and the passband was filtered between 1 and 100 Hz. Then, statistical analysis was performed on the test reports on the amplitude of N2–P2 and the latency of P2 wave.

## Co-IP

120 μL magnetic beads and 15 μg IgG/TSP2 antibody were added to two 1 mL centrifuge tubes respectively, then the two centrifuge tubes were balanced to 500 mL with buffer, placed on a vertical shaker and incubated for 2 h. The unbound antibody was washed away by GLB. Then, the retinal protein lysate was added to the mixture of magnetic beads and antibodies, placed on a vertical shaker and incubated at 4 ℃ for 24 h. The rest of the protein lysate was washed away, then added 1 × loading buffer 40–50 μL, boiled for 5 m, centrifuged for 10000r × 5 m, took the supernatant, run the gel, transferred the membrane, and incubated with the α2δ1(1:200)/IgG(1:1000)/TSP2 (1:500) antibody to develop.

### Image analysis

**Fig. 1:**
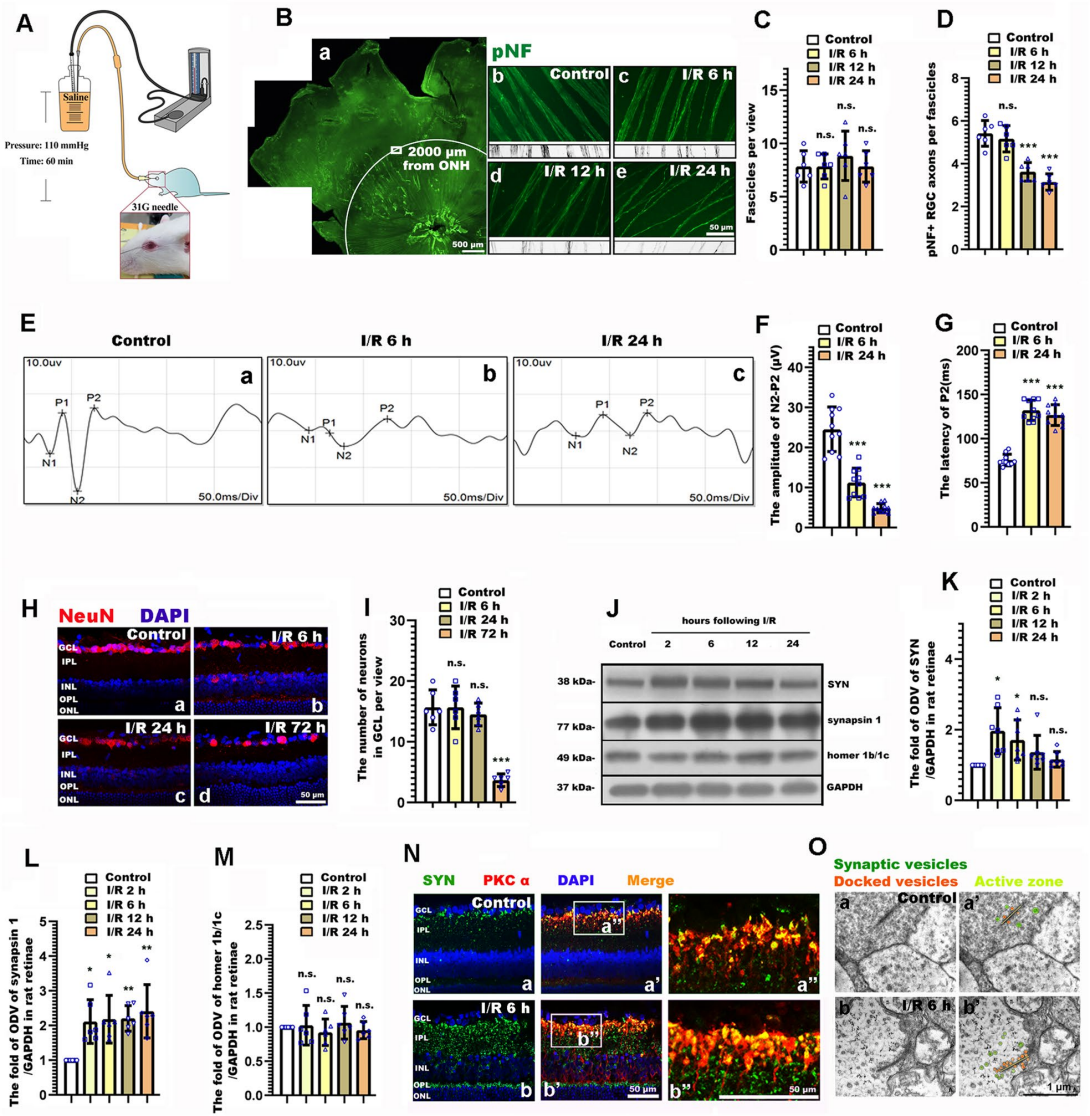
The spatiotemporal profile underling retinal neurite, synaptic alteration, and visual dysfunction following retinal ischemia/reperfusion (RI/R). **A** Diagram of RI/R model. **B** Immunofluorescence images showed the morphological alteration of RGCs axons following RI/R. **B** a Schematic diagram on the selection of views for retinal whole mounts. **C**, **D** Bar graphs depicted the number of fascicles per view and the number of pNF + RGC axons per fascicle in each group(n = 6). **E** Visual dysfunction following RI/R were measured by f-VEP test. **F**, **G** Bar graphs depicted the amplitude of N2-P2 and the latency of P2 in each group (n = 10). **H** Immunofluorescence images showed the neuronal loss in GCL of rat retinae following RI/R. **I** Bar graphs depicted the number of neurons in GCL of rat retinae in each group (n = 6). **J** Western blotting showed the changes in expression of synaptophysin (SYN), synapsin 1 and homer 1b/1c in rat retinae following RI/R. **K**–**M** Bar graphs depicted the fold of optical density value (ODV) of SYN, synapsin 1 and homer 1b/1c in each group(n = 6–7). **N** Double-immunofluorescence images showed spatial location and origin of the increased SYN following RI/R. **O** Synaptic micromorphology was determined by transmission electron microscopy. Green circles: synaptic vesicles, Orange circles: docked vesicles, Yellow strips: active zone. ONH: optical nerve head; GCL: ganglion cell layer; IPL: inner plexiform layer; INL: inner nuclear layer; OPL: outer plexiform layer; ONL: outer nuclear layer. Data in (**C**, **D**,** F**–**G**, **I**, **K**–**M**) were represented as mean ± SD; *p < 0.05, ** *p* < 0.01, *** *p* < 0.001, n.s.: no significance, (compared with the control group using one-way analysis of variance). Bar = 50 μm/500 μm in (**B**), Bar = 50 μm in (**H** and **N**), Bar = 1 μm in (**O**)

The density of RGC axon fasciculation was analyzed in retinal whole mounts immunostained for pNF. Confocal images were projected at maximal intensity to span the entire NFL, and they were pseudocolored and zoomed 200 × to facilitate the visualization of the RGC axons. Retinal sectors with preserved axon fasciculation were selected in each retinal quadrant, a 220 μm × 160 μm view was selected at an eccentricity of 2000 μm from the ONH. For counting the fascicles per view, a 220-μm-long line was traced perpendicular to the fascicles within the healthiest quadrant of each retina. The mean number of pNF^+^ RGC axons per fascicle was counted along each line. Counts included axons bundled together in a fascicle, as well as defasciculated, single axons (Fig. 1b).

The number, the morphology and the distribution of astrocytes were analyzed in retinal whole mounts and retinal frozen slices immunostained for GFAP. Confocal images were pseudocolored and zoomed 200 × to facilitate the visualization of astrocytes. For counting the number of astrocytes, a 220 μm × 160 μm view was selected in four quadrants per retina at an eccentricity of 2000 μm from the ONH. The mean of these 4 values was used to calculate the mean density of parenchymal astrocytes (cells per mm^2^) in each retina. It was not possible to determine absolute densities of vascular- associated astrocytes because the astrocyte layer in each view was partially occupied by a blood vessel of variable, indeterminate volume. Thus, this assessment excluded ‘vascular-associated astrocytes’, that is, astrocytes associated by either their somas or the end feet of processes with the small blood vessels of the retinal capillary bed sometimes presented in the view. Relative mean gray value and relative positive area of GFAP staining in the retinal whole mounts were measured by ImageJ. The range of astrocytic processes in neuroretina layers was measured in GFAP stained retinal slices by masked observers. The normalized ratio of the range of GFAP + astrocytic processes was defined as the (range of GFAP/neuroretina thickness in control or RI/R group)/(range of GFAP/neuroretina thickness in control group).

For each retinal slice, three photos from central region of retina [24] were captured under a 20 × Objective lens (image field: 220 μm × 160 μm) on a confocal microscope (Zeiss LSM780). The neurons in the GCL were defined by labeling with NeuN (neuron marker) and DAPI in GCLs. Three photos from each central region of retina for GFAP, SYN/PKC-α, and TSP2/GFAP double staining were chosen. The SYN from bipolar cells was defined by colabeling of SYN and PKC-α (bipolar cells marker). The TSP2 from astrocytes was defined by colabeling of TSP2 and GFAP (astrocyte marker). The synaptic vesicles in IPL were evaluated by using a HT7700 transmission electron microscope (Hitachi, Tokyo, Japan), by locating at inner plexiform layer. Vesicles binding with the active zone were defined as docked vesicles [11].

### Data analysis

**Fig. 2:**
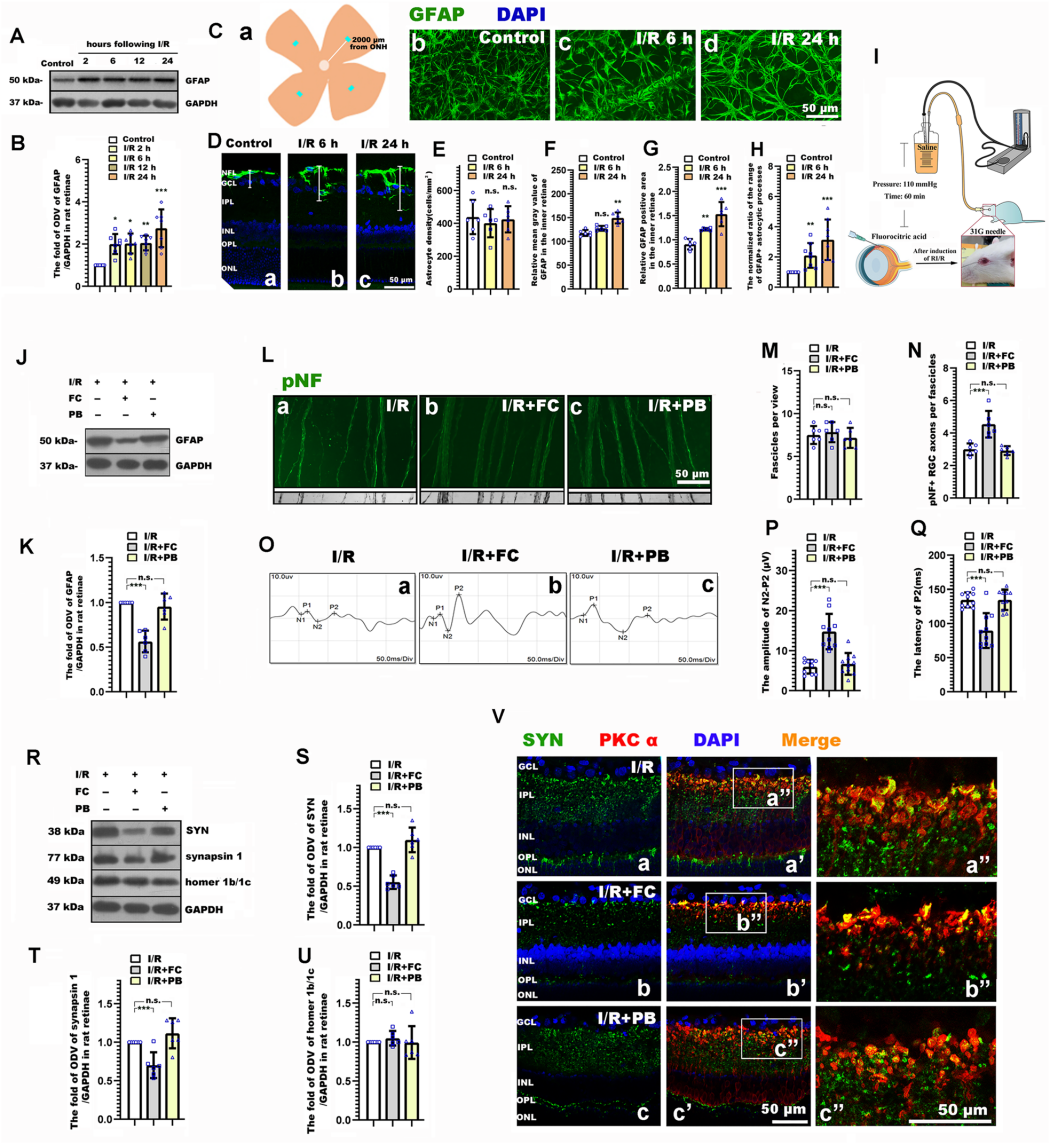
Fluorocitric acid (FC) treatment ameliorated the neurite and synaptic alteration, and the degenerated visual function following RI/R. **A** Western blotting showed the change of GFAP expression in rat retinae following RI/R. **B** Bar graphs depicted the fold of optical density value (ODV) of GFAP in each group (n = 7).** C** Immunofluorescence images showed the number of astrocytes, the spatial location of the increased GFAP. **C** a Schematic diagram on the selection of views for retinal whole mounts. **D** Immunofluorescence images showed the range of GFAP + astrocytic processes following RI/R. **E–G** Bar graphs depicted the density of astrocytes, the relative mean gray value of GFAP and the relative GFAP positive area in the inner retinae in each group (n = 6). **H** Bar graphs depicted the normalized ratio of the range of GFAP + astrocytic processes in each group (n = 6). **I** Diagram of FC treatment after RI/R. **J** Western blotting showed the change of GFAP expression after FC treatment. **K** Bar graphs depicted the fold of optical density value (ODV) of GFAP in each group (n = 6). **L** Immunofluorescence images showed the morphological alteration of RGCs axons with FC treatment following RI/R. **M–N** Bar graphs depicted the number of fascicles per view and the number of pNF + RGC axons per fascicle in each group (n = 6). **O** Visual function following RI/R with FC treatment were measured by f-VEP test. **P**, **Q** Bar graphs depicted the amplitude of N2-P2 and the latency of P2 in each group (n = 10). **R** Western blotting showed the change in expression of synaptophysin (SYN), synapsin 1 and homer 1b/1c in rat retinae with FC treatment following RI/R. **S**–**U** Bar graphs depicted the fold of optical density value (ODV) of SYN, synapsin 1 and homer 1b/1c in each group (n = 6). **V** Double-immunofluorescence images showed spatial location and origin of SYN in each group with FC treatment following RI/R. Data in (**B**, **E**–**H**) were represented as mean ± SD; **p < 0.01, *** *p* < 0.001, n.s.: no significance, (compared with the control group using one-way analysis of variance). Data in **K**, **M**, **N**, **P**, **Q**, **S**–**U** were represented as mean ± SD; *p < 0.05, **p < 0.01, *** *p* < 0.001, n.s.: no significance, (compared with the RI/R group using one-way analysis of variance). Bar = 50 μm in (**C**, **D**, **L**, **V**). Abbreviations are as defined in Fig. 1

FluorChem8900 software was used to analyze the optical density value (ODV) of the bands of GFAP/SYN/synapsin 1/homer 1b/1c/TSP2/α2δ1 by western blotting. In detail, the selected image was opened, then the background was subtracted, frame selected the lanes, and recorded the ODV of each lane. The average values of GFAP/SYN/synapsin 1/homer 1b/1c/TSP2/α2δ1 and GAPDH were compared, and the average relative values were obtained. In experiments that the animals only received RI/R injury, we set these average relative values in control group to 1, and the fold relative values for other I/R groups were obtained and analysis after being compared with the average relative values in the control group (e.g., Fig. 1K). In experiments that animals are treated with FC/TSP2 shRNA/GBP before or after RI/R injury, we set these average relative values in I/R groups to 1, and the fold relative values for I/R + FC/TSP2 shRNA/GBP group and I/R + PB/Scramble RNA were obtained and analyzed after being compared with average relative values in I/R group (e.g., Fig. 2R). One-way analysis of variance and Bonferroni’s Multiple Comparison Test was performed to test differences between groups. All the results are presented as the mean ± standard deviation of results from at least three independent experiments. All statistical analyses were performed on GraphPad Prism 8.0 (GraphPad Software, La Jolla, CA, USA). A value of *p* < 0.05 was considered as statistically significant and the significance is indicated in the graphs by an asterisk, and *P* values less than 0.01 and 0.001 are indicated by two and three asterisks, respectively. Non-significance (n.s.) indicates no significant changes.

## Results

### RGC axons degeneration, the visual dysfunction and synaptic remodeling in IPL preceded the neuronal loss in GCL following RI/R

Retinal ganglion cell (RGC) axons connect the eyes to the brain through delivering visual signals, fail to regenerate after damage, eventually leading to visual dysfunction [25]. In this part of the experiment, we firstly observed the change of RGC axons following RI/R. Phosphorylated neurofilament (pNF), a cytoskeletal marker extensively used to selectively track RGC axons [26, 27], was introduced to evaluate the integrity of axon fasciculation in NFL following RI/R. Healthy retinae (control group) displayed RGC axons uniformly segregated in tightly packed fascicles, RGC axons in I/R groups exhibited defasciculation, with fascicle thinning, even solitary axons (Fig. 1B). Statistical analysis showed that the number of fascicles per view presented no obvious difference between the control group and I/R groups, while, pNF + axons per fascicle significantly decreased from 6 h following RI/R (Fig. 1C, D). VEP latency and amplitude reflects the velocity of signal along the visual pathway and the axonal degeneration of RGCs respectively [28, 29]. Therefore, the rats were subjected to f-VEP test at 6 h and 24 h post trauma [axonal changes of RGCs emerged within 6 h following RI/R (Fig. 1B–D)]. Results showed that the amplitude of P2 significantly decreased within 6 h following RI/R, and the latency of P2 extended (Fig. 1E–G). These events were temporally consistent with the defasciculation of RGC axons. However, immunofluorescence images revealed that no obvious decrease of NeuN + cells in GCL emerged until 72 h following RI/R. (Fig. 1H, I). The above results suggest that the RGC axon degeneration and the visual dysfunction emerged within 24 h following RI/R, which preceded the neuronal loss in GCL.

We next explored the specific pattern of synaptic remodeling in IPLs where the dendrites of ganglion cells mainly form synapses with the axons of bipolar cells, relaying visual signals from photoreceptors [30]. The synaptic remodeling-related protein (synaptophysin (SYN), synapsin 1, and homer 1b/1c) were measured after RI/R treatment. We found that the protein level of SYN and synapsin 1, associated with synaptic vesicle exocytosis, significantly increased from 2 h post trauma (Fig. 1J–L). However, homer 1b/1c (a vital component of postsynaptic densities, which form the basis of synaptic transmission) [31] remained unchanged (Fig. 1J, M). These data implied that presynaptic proteins significantly increased at the early stage following RI/R injury, while there was no change of postsynaptic proteins. For further observation, immunofluorescence showed that the increased SYN was mainly expressed in the inner plexiform layer (IPL) (Fig. 1Na, Nb). Co-labeling with PKCα, a bipolar cell marker, revealed that bipolar cells in the innermost IPL were among the cells with upregulated SYN. (Fig. 1Na″, Na″, Nb′, Nb″). By using transmission electron microscopy, we found that more docked vesicles and synaptic vesicles per synapse presented following RI/R (Fig. 1Oa′, Ob′). Based on these results, we confirmed that RI/R increased presynaptic proteins, docked vesicles and synaptic vesicles per synapse between RGCs and bipolar cells in the IPL of rat retinae early post trauma. These increases occurred in bipolar cells at the innermost IPL where bipolar cells synapse with RGCs. While, there was no response from postsynaptic components.

### Suppressing astrocytic activation ameliorated RI/R induced neurite and synaptic alteration, and the degenerated visual function

We firstly tested the protein level of GFAP (astrocytic activation marker) following RI/R. Western blotting showed that the expression of GFAP significantly increased following RI/R (Fig. 2A, B). Meanwhile, the astrocytic processes in rat retinae became thicker and longer, and the extension area of single astrocytic processes obviously increased (Fig. 2C). Image analysis revealed the relative mean gray value of GFAP, the relative GFAP positive area (Fig. 2F, G). However, the number of astrocytes remained unchanged (Fig. 2E). Furthermore, Immunofluorescence and the image analysis revealed that the normalized ratio of the range of GFAP + astrocytic processes significantly increased after I/R treatment (Fig. 2D, H). These results suggested that although the number was not changeable, astrocytes presented an obvious activation and a morphological change following RI/R. Besides, the astrocytic processes in rat retinae tended to extend into the outer retinae after I/R treatment. These events were similar to the temporal pattern of RGC axons degeneration and synaptic remodeling in IPL (Fig. 1). The above evidence implied that these activated astrocytes might be associated with the neurite and synaptic alteration following RI/R.

Fluorocitrate acid (FC), which is preferentially taken up by astrocytes, blocks the production of tricarboxylic acid cycle intermediates and transiently suppresses their signaling activity [32]. With a strict dosage control, the local administration of FC can effectively suppress the activation of astrocytes, while it has no effect on surrounding neurons and astrocytes in normal tissues [33, 34]. Thus, we intravitreally injected FC to suppress the activation of astrocytes to explore the potential role of activated astrocyte underlying the synaptic and neurite changes following RI/R. We found that while RI/R induced high protein levels of GFAP, these were significantly decreased following intravitreal injection of FC (Fig. 2J, K), suggesting the activation of astrocyte was effectively suppressed. Thus, FC was used in the subsequent experimental evaluations: By immunofluorescence, we found that RGC axons represented a uniform separation in tightly packed fascicles following intravitreal injection of FC. (While, RGC axons in I/R group and I/R + PB group exhibited defasciculation, with fascicle thinning.) Statistical analysis showed that pNF + axons per fascicle in I/R + FC group significantly increased as compared with the I/R and I/R + PB groups (Fig. 2L–N). F-VEP tests revealed increased amplitudes of P2, and a significantly shortened P2 latency following intravitreal injection of FC (Fig. 2O–Q). Furthermore, Western blotting showed that the protein level of SYN and synapsin 1 in the I/R + FC group was significantly lower than that in the I/R group and I/R + PB groups. This difference was not found when evaluating the expression of homer 1b/1c. (Fig. 2R–U). Double-immunofluorescence revealed that the RI/R induced up-regulated presynaptic protein in bipolar cells decreased following FC treatment. (Fig. 2Va′, Va″, Vb′, Vb″, Vc′, Vc″). These evidences demonstrate that activated astrocytes might contribute to the RI/R induced neurite and synaptic alterations, ultimately causing visual dysfunction.

### Astrocyte derived TSP2 participated in neurite and synaptic alteration, and the degenerated visual function following RI/R

**Fig. 3:**
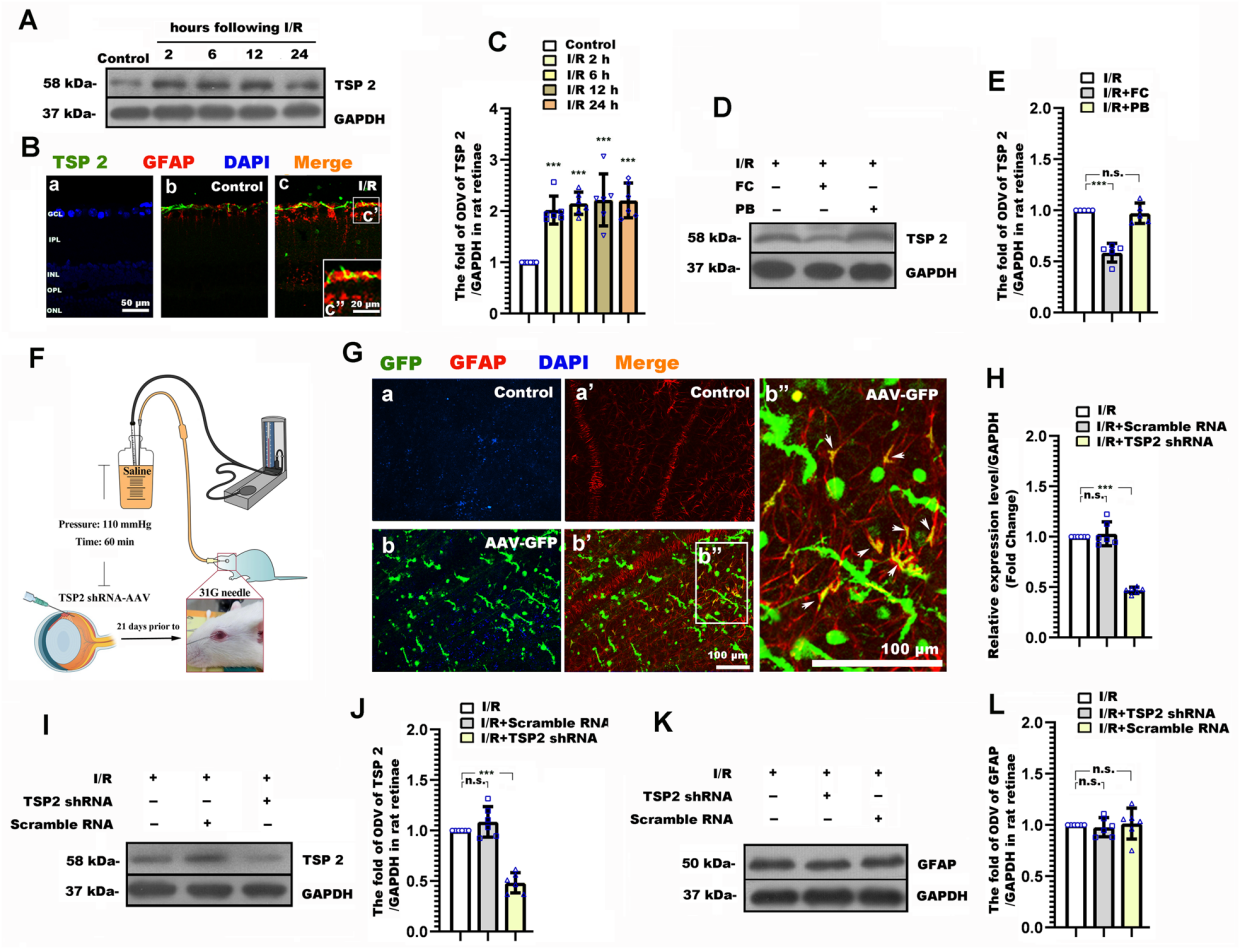
Thrombospondin2 (TSP2) shRNA-adeno-associated virus (AAV) treatment suppressed the expression of TSP2 in rat retinae following RI/R. **A** Western blotting showed the change of TSP2 expression in rat retinae following RI/R. **B** Double-immunofluorescence images revealed the origination of TSP2. **C** Bar graphs depicted the fold of optical density value (ODV) of TSP2 in each group (n = 6). **D** Western blotting showed the change of TSP2 expression in rat retinae with the FC treatment following RI/R. **E** Bar graphs depicted the fold of optical density value (ODV) of TSP2 in each group compared to I/R group (n = 5). **F** Diagram of TSP2 shRNA AAV treatment before RI/R. **G** Fluorescence images depicted the transduction of AAV following intravitreal injection of AAV-GFP-TSP 2 shRNA. Co-staining with GFAP revealed that the TSP2 shRNA was expressed in astrocytes in rat retina (shown by the white arrows). **H** QPCR showed the change of TSP2 mRNA level in rat retinae with the TSP2 shRNA-AAV treatment following RI/R (n = 6). **I** Western blotting showed the change of TSP2 expression in rat retinae with the TSP2 shRNA-AAV treatment following RI/R. **J** Bar graphs depicted the fold of optical density value (ODV) of TSP2 in each group compared to I/R group (n = 6). **K** Western blotting showed the change of GFAP expression in rat retinae with the TSP2 shRNA-AAV treatment following RI/R. **L** Bar graphs depicted the fold of optical density value (ODV) of GFAP in each group (n = 6). Data in (**C**) were represented as mean ± SD; *** *p* < 0.001, (compared with the control group using one-way analysis of variance). Data in (**E**, **J**, **L**) were represented as mean ± SD; ***p < 0.001, n.s.: no significance, (compared with the RI/R group using one-way analysis of variance). Bar = 50 μm/20 μm in (**B**), Bar = 100 μm in (**G**). The abbreviations are as defined in Fig. 1

**Fig. 4:**
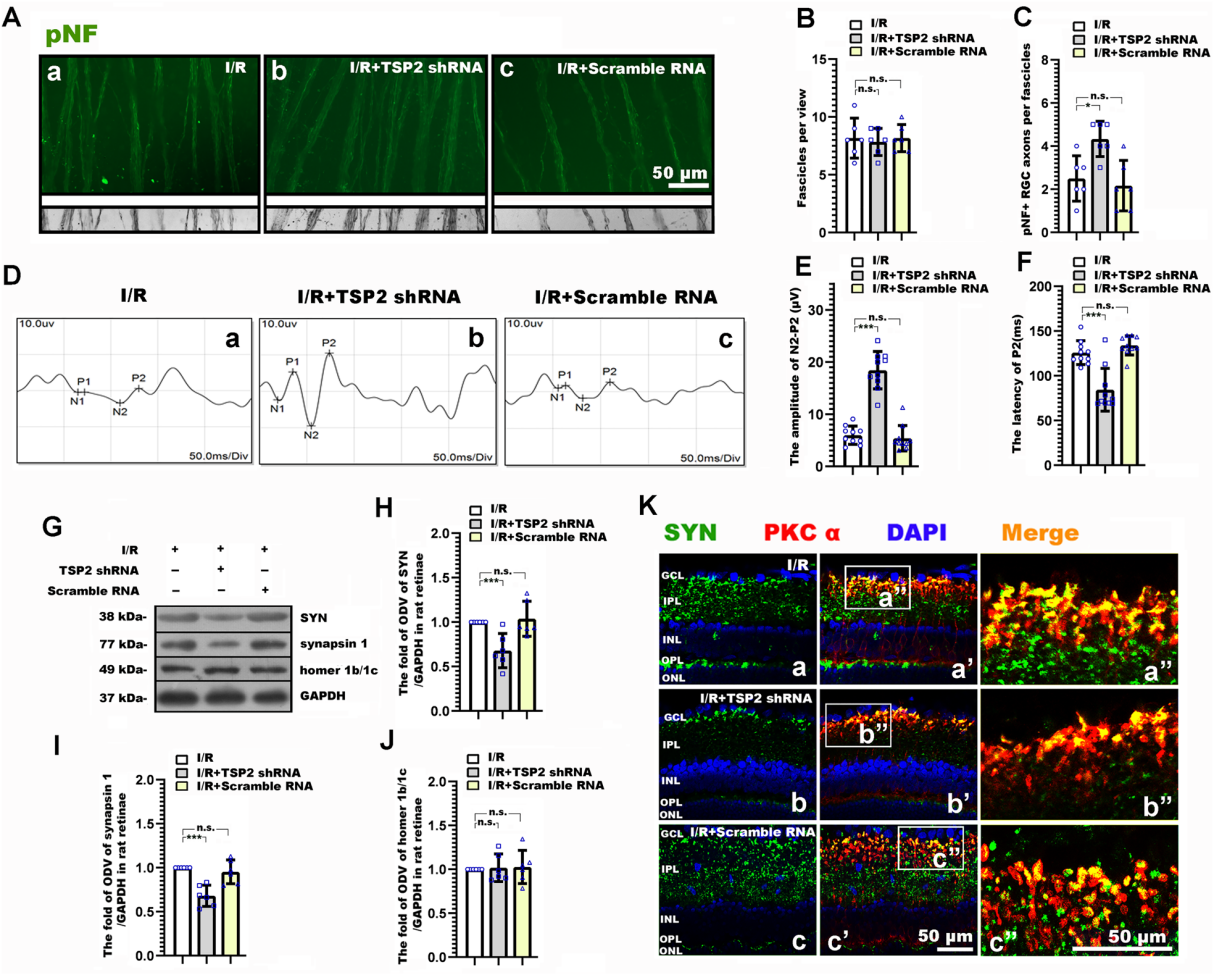
TSP2 shRNA-AAV treatment ameliorated the neurite and synaptic alteration, and the degenerated visual function following RI/R. **A** Immunofluorescence images showed the morphological alteration of RGCs axons with TSP2 shRNA-AAV treatment following RI/R. **B**, **C** Bar graphs depicted the number of fascicles per view and the number of pNF + RGC axons per fascicle in each group (n = 6). **D** Visual function following RI/R with TSP2 shRNA-AAV treatment were measured by f-VEP test. **E**, **F** Bar graphs depicted the amplitude of N2-P2 and the latency of P2 in each group (n = 10). **G** Western blotting showed the change in expression of synaptophysin (SYN), synapsin 1 and homer 1b/1c in rat retinae with TSP2 shRNA-AAV treatment following RI/R. **H**, **J** Bar graphs depicted the fold of optical density value (ODV) of SYN, synapsin 1 and homer 1b/1c in each group (n = 6). **K** Double-immunofluorescence images showed spatial location and origin of SYN in each group with TSP2 shRNA-AAV treatment following RI/R. Data in (**B**, **C**, **E**, **F**, **H**, **J**) were represented as mean ± SD; *p < 0.05, **p < 0.01, n.s.: no significance, (compared with the RI/R group using one-way analysis of variance). Bar = 50 μm in (**A**, **K**). The abbreviations are as defined in Fig. 1

It has been proven that the astrocyte-derived TSP2 mediated synaptogenesis and synaptic remodeling during CNS development and injuries [35, 36]. We previously found that the increased TSP2 occurred early following elevated hydrostatic pressure in vitro, which was temporally consistent with the increased presynaptic components [20]. Thus, we further explored the potential roles of astrocyte-derived TSP2 in RI/R induced RGC neurite degeneration, synaptic alteration and visual dysfunction. The expression of TSP2 in the rat retina was tested at 2 h, 6 h, 12 h and 24 h following RI/R. Western blotting showed that the TSP2 expression began to upregulate at 2 h post trauma (Fig. 3A, C). Moreover, immunofluorescence revealed that the increased TSP2 was co-labeled with GFAP (Fig. 3B), which suggested that the increased TSP2 was mainly derived from activated astrocytes. To determine the specific role of astrocyte-derived TSP2 in neurite and synaptic alteration, and visual dysfunction following RI/R, TSP2 shRNA-AAV was transduced through intravitreal injection. AAV-GFP showed that AAV were successfully transduced into cells in rat retinae (Fig. 3Ga, Gb), and the confocal images showed that TSP2-shRNA GFP was co-existed in astrocytes following co-staining with GFAP (shown in Fig. 3Gb′ and by the white arrows in Fig. 3Gb″). These evidences suggested that the TSP 2 shRNA was expressed in the astrocytes. QPCR and Western blotting suggested that RI/R induced high expression of TSP2 significantly decreased by intravitreal injection of TSP2 shRNA-AAV. (Fig. 3H–J). Notably, the RI/R induced upregulated expression of TSP2 could be suppressed by FC (Fig. 3D–E), while TSP2 shRNA-AAV transduction could not downregulate the high expression of GFAP following RI/R (Fig. 3K, L). These data implied that the activated astrocytes could upregulate TSP2 expression following RI/R injury, but the change of TSP2 could not influence the astrocytic activation. In this scenario, we chose TSP2 shRNA-AAV for the following experiments: By immunofluorescence, we found that knockdown TSP2 showed that RGC axons represented as approximately uniform separation in tightly packed fascicles (Fig. 4A). Statistical analysis showed that the number of fascicles per view present pNF + axons per fascicle in I/R + TSP2 shRNA group significantly increased as compared with the I/R and I/R + scramble RNA groups (Fig. 4B, C). F-VEP tests showed that the amplitudes of P2 increased, and the P2 latency was shortened to a certain extent following intravitreal injection of TSP2 shRNA-AAV (Fig. 4D–F). Furthermore, the protein level of SYN and synapsin 1 in the I/R + TSP2 shRNA group was significantly lower than that in the I/R and I/R + scramble RNA groups (Fig. 4G–I). This difference was not found when evaluating the expression of homer 1b/1c. (Fig. 4G, J). Double-immunofluorescence revealed that the RI/R induced up-regulated presynaptic protein in bipolar cells decreased following TSP2 shRNA-AAV treatment. (Fig. 4Ka′, Ka″, Kb′, Kb″, Kc′, Kc″). These evidences demonstrated that activated astrocytes-derived TSP2 might contribute to the RI/R induced neurite and synaptic alteration and visual dysfunction.

**Fig. 5:**
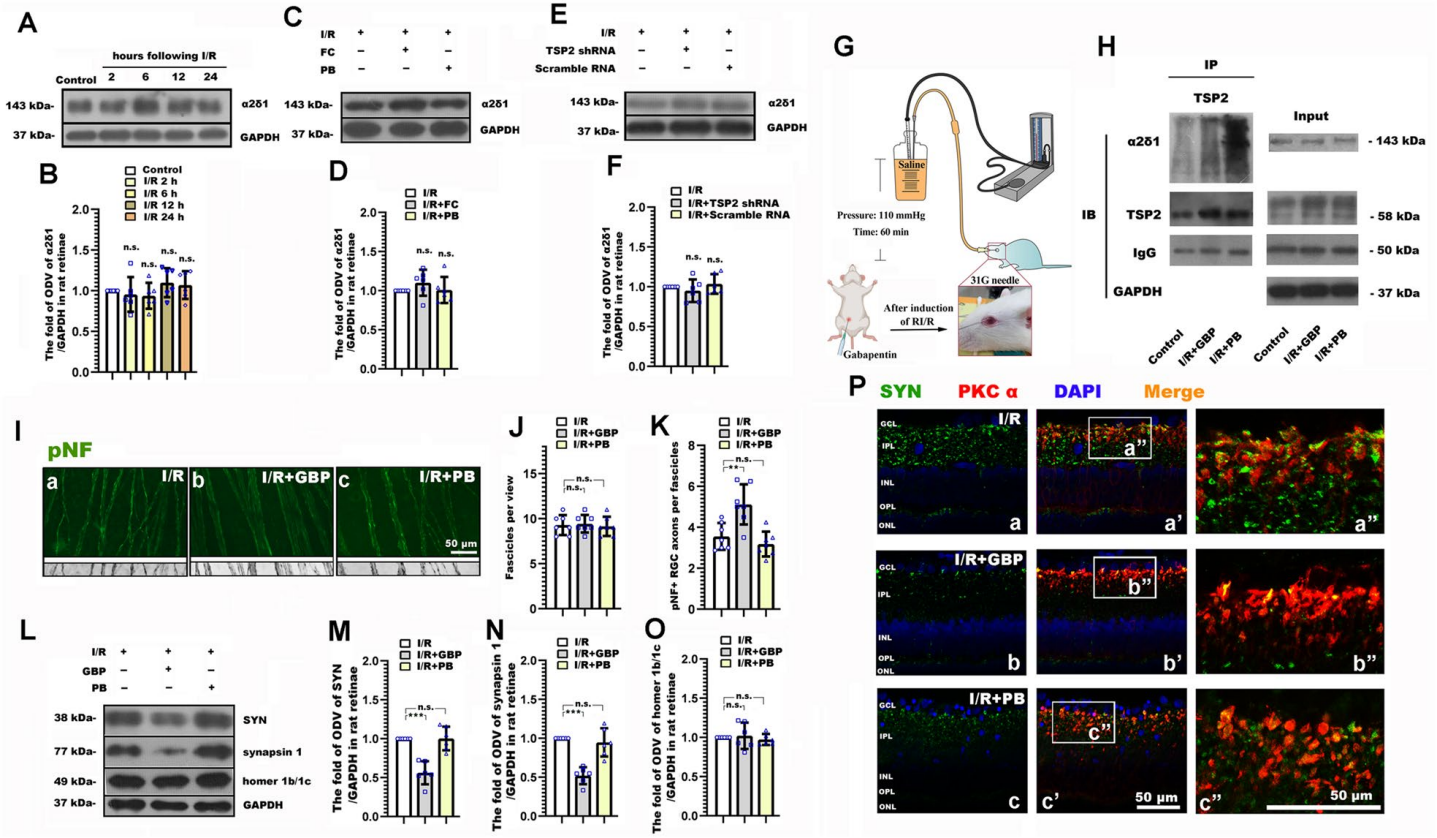
GBP treatment ameliorated the neurite and synaptic alteration, and the degenerated visual function following RI/R. **A** Western blotting showed the change of α2δ-1 expression in rat retinae following RI/R. **B** Bar graphs depicted the fold of optical density value (ODV) of α2δ-1 in each group (n = 6). **C** Western blotting showed the change in expression of α2δ-1 in rat retinae with FC treatment following RI/R. **D** Bar graphs depicted the fold of optical density value (ODV) of α2δ-1in each group (n = 6). **E** Western blotting showed the change in expression of α2δ-1 in rat retinae with TSP2 shRNA-AAV treatment following RI/R. **F** Bar graphs depicted the fold of optical density value (ODV) of α2δ-1in each group. (n = 6). **G** Diagram of GBP treatment before RI/R. **H** Co-immunoprecipitation depicted the binding of TSP2 and α2δ-1 in rat retinae with GBP treatment following RI/R. **I** Immunofluorescence images showed the morphological alteration of RGCs axons with GBP treatment following RI/R. **J**, **K** Bar graphs depicted the number of fascicles per view and the number of pNF + RGC axons per fascicle in each group (n = 7). **L** Western blotting showed the change in expression of synaptophysin (SYN), synapsin 1 and homer 1b/1c in rat retinae with GBP treatment following RI/R. **M**–**O** Bar graphs depicted the fold of optical density value (ODV) of SYN, synapsin 1 and homer 1b/1c in each group (n = 6). **P** Double-immunofluorescence images showed spatial location and origin of SYN in each group with GBP treatment following RI/R. Data in (**B**) were represented as mean ± SD; n.s.: no significance, (compared with the control group using one-way analysis of variance). Data in (**D**, **F**, **J**, **K**, **M**–**O**) were represented as mean ± SD; *p < 0.05, **p < 0.01, ***p < 0.001, n.s.: no significance, (compared with the RI/R group using one-way analysis of variance). Bar = 50 μm in (**I**, **P**). The abbreviations are as defined in Fig. 1

In order to determine whether TSP2 mediates the neurite and synaptic alteration following RI/R through binding to α2δ1, Gabapentin (GBP) [37] was used to competitively inhibit the combination of TSP2 and α2δ1. Western blotting showed that the expression of α2δ1 presented no significant difference between the control and I/R groups. (Fig. 5A, B). Furthermore, there was no difference in α2δ1 expression by FC and TSP2 shRNA-AAV treatment. (Fig. 5C–F). These data implied that RI/R injury, suppressing astrocytic activation, and downregulating TSP2 expression could not affect the expression of α2δ1. Co-immunoprecipitation (Co-IP) revealed that the binding rate of TSP2 and α2δ1 increased following RI/R, which could be significantly decreased by GBP treatment. (Fig. 5H). Therefore, we used GBP to perform the subsequent experiments: Immunofluorescence displayed RGC axons in I/R + GBP group uniformly segregated in tightly packed fascicles, RGC axons in I/R group and I/R + PB group exhibited defasciculation, with fascicle thinning (Fig. 5I). Statistical analysis showed that pNF + axons per fascicle in I/R + GBP group increased as compared with the I/R and I/R + PB groups (Fig. 5J, K). Moreover, the protein level of SYN and synapsin 1 in the I/R + GBP group was significantly lower than that in the I/R and I/R + PB groups (Fig. 5L–N). This difference was not found when evaluating the expression of homer 1b/1c. (Fig. 5L, O). Furthermore, double-immunofluorescence showed that the RI/R induced up-regulated presynaptic protein in bipolar cells decreased following GBP treatment. (Fig. 5Pa′, Pa″, Pb′, Pb″, Pc′, Pc″).

In summary, our findings provide corroborating evidences that the astrocyte-induced “TSP2-α2δ1” pathway may participate in the neurite and synaptic alteration following RI/R, which can ultimately lead to visual dysfunction.

## Discussion

### Neurite degeneration, visual dysfunction and synaptic alteration emerge at the early stage after RI/R injury

In this study, we used morphological and molecular methods to evaluate the detailed profile of neurite and synaptic alteration following retinal ischemia/reperfusion injury. We found that as early as 6 h after RI/R injury, although the number of neurite fascicles didn’t significantly change, pNF + RGC axons presented as defasciculation, even solitary (Fig. 1B). This pathological event will certainly hinder the visual signals being transmitted to the lateral geniculate nucleus (LGN) and the superior colliculus (SC). Indeed, f-VEP reports showed that the degeneration of visual function emerged at 6 h after RI/R injury (Fig. 1E–G). These data suggest that the alteration of RGC axons and the visual dysfunction may occur in the early stage after RI/R injury, which precedes the neuronal loss in the ganglion cell layer (GCL) (Fig. 1H, I). Addressing the need for amelioration of the RI/R induced visual dysfunction, even recovery of the visual acuity, the above evidences told us that attention should be paid to the earlier changes of RGC axons after RI/R injury. This comes in contrast with past hypotheses, often focusing on the loss of neurons in GCL [5, 6].

Our further exploration revealed that there were some interesting transneuronal alterations in the inner layers of rat retinae in the early stage after RI/R injury. Specifically, synaptic vesicle proteins in the inner plexiform layer (IPL) of the rat retina increased from 6 h after RI/R injury, while there was no difference when evaluating the post-synaptic components (Fig. 1J–N). It is worth paying attention to synapses between RGCs and bipolar cells following RI/R injury, since the main neurite degeneration and cell death occur in the RGCs. In the present study, we found that presynaptic proteins in the bipolar cells, analyzed by SYN, increased in the IPL early after RI/R injury (Fig. 1N), which is similar with the temporal pattern of RGC axonal degeneration and visual dysfunction. Furthermore, compared with the control group, RI/R injury induced a greater number of docked and synaptic vesicles per ribbon synapse between RGCs and bipolar cells (Fig. 1O). These features indicated newly formed, but immature synapses in IPL, which might suggest a compensatory mechanism to maintain/restore the synaptic connections between RGCs and bipolar cells due to the degeneration of RGC axons. Consistent with our findings, Hae-Young Loilly Park et al. revealed that the immature synapse was formed accompanied by gradual loss of RGCs due to chronic intraocular pressure (IOP) elevation [11]. Previous studies showed that these early newborn synapses are usually established rapidly after injury. Then the distal neurons could be affected by changing the expression level of synaptic active protein [38], adjusting the level of cytokines [39, 40], or by altering the content of mitochondria to transmit damage signals [41]. These are the transneuronal compensatory responses to injury signals [39]. Taken together, we can demonstrate that the early degeneration of RGC axons in our study impedes the visual signals transduction following RI/R injury. In this scenario, bipolar cells may try to increasingly synapse with RGCs to maintain, even recovery the visual function by up-regulating synaptic vehicle proteins. However, the postsynaptic protein, analyzed by homer 1b/1c, presented no difference in this case (Fig. 1J, M), which suggests that the mature synapses have not been formed. Research from neuroscience suggests: promoting neurogenesis may be a compensatory response for brain repair mechanism, but dysfunctional neurogenesis resulting from early disease manifestations may in turn exacerbate neuronal vulnerability to the insults [42]. In the present study, the increased synaptic vehicle proteins and presynaptic vesicles might induce excitotoxic neurotransmitters release and excessive energy consumption [43] in bipolar cells. This may increase the bipolar cells’ susceptibility to the RI/R injury. However, these speculations need to be further explored.

### “Astrocyte-TSP2-α2δ1” pathway contributes to neurite degeneration, synaptic alteration and visual dysfunction following RI/R

In order to explore the potential role of activated astrocytes in RI/R induced RGC neurite degeneration, synaptic alteration and visual dysfunction, we inhibited the activation of astrocytes through intravitreal FC injection. The subsequent experiments demonstrated that the RGC axons defasciculation, the compensatory response of synaptic components in IPL and the visual dysfunction were all efficiently alleviated by FC treatment (Fig. 2). It has been proven that astrocytes undergo a pronounced transformation in morphology, transcriptional profile and function, called reactive astrogliosis following CNS diseases and injuries [44]. In this process, astrocytes can be polarized into dual neurotoxic (A1) and neuroprotective (A2) functional phenotypes [44, 45]. A1 astrocytes upregulate the initial part of the classical complement cascade (C1r, C1s, C3, and C4), which previously has been shown to be harmful to synapse [44, 45]. On the contrary, A2 astrocytes upregulate many neurotrophic factors (CLCF1, LIF, and IL6), which promote neuronal survival [44–46]. FC has been shown that could ameliorate the A1 functional phenotypic polarization of astrocytes [34]. Furthermore, the down-regulation of synaptic vehicle proteins might lessen the excitotoxic neurotransmitters release and neuronal energy consumption. These might be the reasons that the RGC axonal degeneration and the visual dysfunction were ameliorated through effectively suppressing astrocytic activation following RI/R.

In order to inhibit the deleterious effects of astrocytes without affecting its beneficial effects for neurons, we need to further explore the potential mechanisms of astrocytes in RI/R induced RGC neurite degeneration, synaptic alteration and visual dysfunction. Previous studies revealed that astrocyte-derived TSP2 participated in synaptogenesis and promoted the excitotoxic neurotransmitters release [35, 36]. Under stroke, TSP2 obviously increased at the ischemia foci, which mainly co-located with astrocytes [47]. We also found that the up-regulation of astrocyte derived TSP2 emerged in rat retinae at 2 h after RI/R injury (Fig. 3A–C). Subsequent experiments showed that FC suppressed the expression of retinal TSP2 following RI/R injury, which suggested that the activation of astrocytes might be one of the key reasons for the up-regulation of TSP2 in this case (Fig. 3D, E). Christopherson KS et al. illustrated that knockdown TSP2 from astrocytes could efficiently restrain CNS synaptogenesis and the excitotoxic neurotransmitters release [36]. Thus, in the present study, we used TSP2 shRNA AAV to knockdown TSP2 in rat retinae. Through AAV-GFP and further co-labeling with GFAP, we confirmed that the TSP2 shRNA was successfully expressed in astrocytes (Fig. 3Gb, Gb′, Gb″). Through qRT-PCR and western blotting, we assessed the efficiency of TSP 2 knockdown (Fig. 3H–J). Data from Fig. 4 suggested that selectively knockdown TSP2 could alleviate the RGC axons defasciculation, the compensatory response of synaptic components in IPL and the visual dysfunction. These pieces of evidences concluded that the up-regulation of TSP2 might be one of the key mechanisms for astrocytes contributing to RI/R induced RGC neurite degeneration, synaptic alteration and visual dysfunction.

Eroglu et al. previously found that TSP2 could bind with α2δ1, a subunit of voltage-gated calcium channels on neuronal membrane, to induce Ca^2+^ influx, reorganization of cytoskeleton proteins, aggregation of synaptic adhesion molecules and synaptogenesis [48]. Consistent with those observations, we presently found that the binding rate of TSP2 and α2δ1 increased following RI/R (Fig. 5H). Besides TSP2, α2δ1 is also present as the high-affinity receptor for gabapentin and pregabalin [49]. Gabapentin (GBP) and pregabalin (PGB) can pass through the blood–brain barrier (BBB) and blood-retina barrier (BRB) to competitively bind with α2δ1, keep α2δ1 in its inactive closed state. In this scenario, TSP2 cannot bind to α2δ1 to promote synaptogenesis [50]. Further research showed that α2δ1 is required for the ability of gabapentin to reduce excitotoxic neurotransmitters release in neuronal tissue, consistent with a therapeutic mechanism of action via voltage-gated calcium channels [51]. In the current study, we intraperitoneally injected GBP to lessen the binding rate of TSP2 and α2δ1. Co-immunoprecipitation (Co-IP) revealed that the binding rate of TSP2 and α2δ1 could be significantly decreased by GBP treatment following RI/R injury (Fig. 5H). In this case, RGC axons defasciculation and the compensatory response of synaptic components in IPL were alleviated to some extent (Fig. 5I–P). The above evidences demonstrated that following RI/R injury, astrocytes were activated to release TSP 2, which bounded with α2δ1 on neuronal membrane, then triggered the early RGC axonal degeneration and the early compensatory response of synaptic components in IPL. This might be the potential mechanism for visual dysfunction that emerges at the early stage after RI/R injury.

It has been proposed that the binding between the EGF-like repeats of TSP2 and the VWF-A domain of α2δ-1 could cause a conformational change in the molecule from closed to open. Then, the activated α2δ-1 recruits an as yet unidentified signaling partner(s) (binding region unknown) to form a “synaptogenic signaling complex” [52]. This activated TSP2/α2δ-1 complex may nucleate a synaptic adhesion by the recruitment of cell adhesion and scaffolding proteins to the potential synaptic sites, and then induce an intracellular signaling cascade that ultimately leads to synaptogenesis and/or synaptic remodeling. Through deeper analysis of the synapses in α2δ-1KO mice, Eroglu et al. found that the activity of the Rho family of small GTPases might be associated with the synaptogenic effect of TSP2/α2δ-1 complex [53]. Previous research showed that among these GTPases, cell division control protein 42 (Cdc42) and Ras-related C3 botulinum toxin substrate 1 (Rac1) participate in various stages of synaptic development [54, 55]. The subsequent experimental findings of Eroglu et al. demonstrated that after TSP 2 binding, α2δ-1 at the post- but not presynaptic surface brings together pre- and postsynaptic components to form synapses; and the C terminus of α2δ-1 triggers intracellular signaling via GEFs Kalirin-7 or β-Pix to stimulate GTP binding to Rac1, promoting actin reorganization [53]. This intracellular signaling cascade ultimately leads to synaptogenesis and/or synaptic remodeling. Simultaneously, TSP2 reorganizes the extracellular matrix (ECM) by interacting with TGF-β1 as well as regulating the balance between MMPs and TIMPs [56]. This event provides the requisite extracellular space for synaptogenesis and/or synaptic remodeling. In sum, the above proposed synaptogenic/synaptic remodeling signaling machinery might guide the subsequent mechanism exploration of TSP2/α2δ-1 complex contributing to the synaptogenic/synaptic remodeling following RI/R.

## Conclusions

**Fig. 6:**
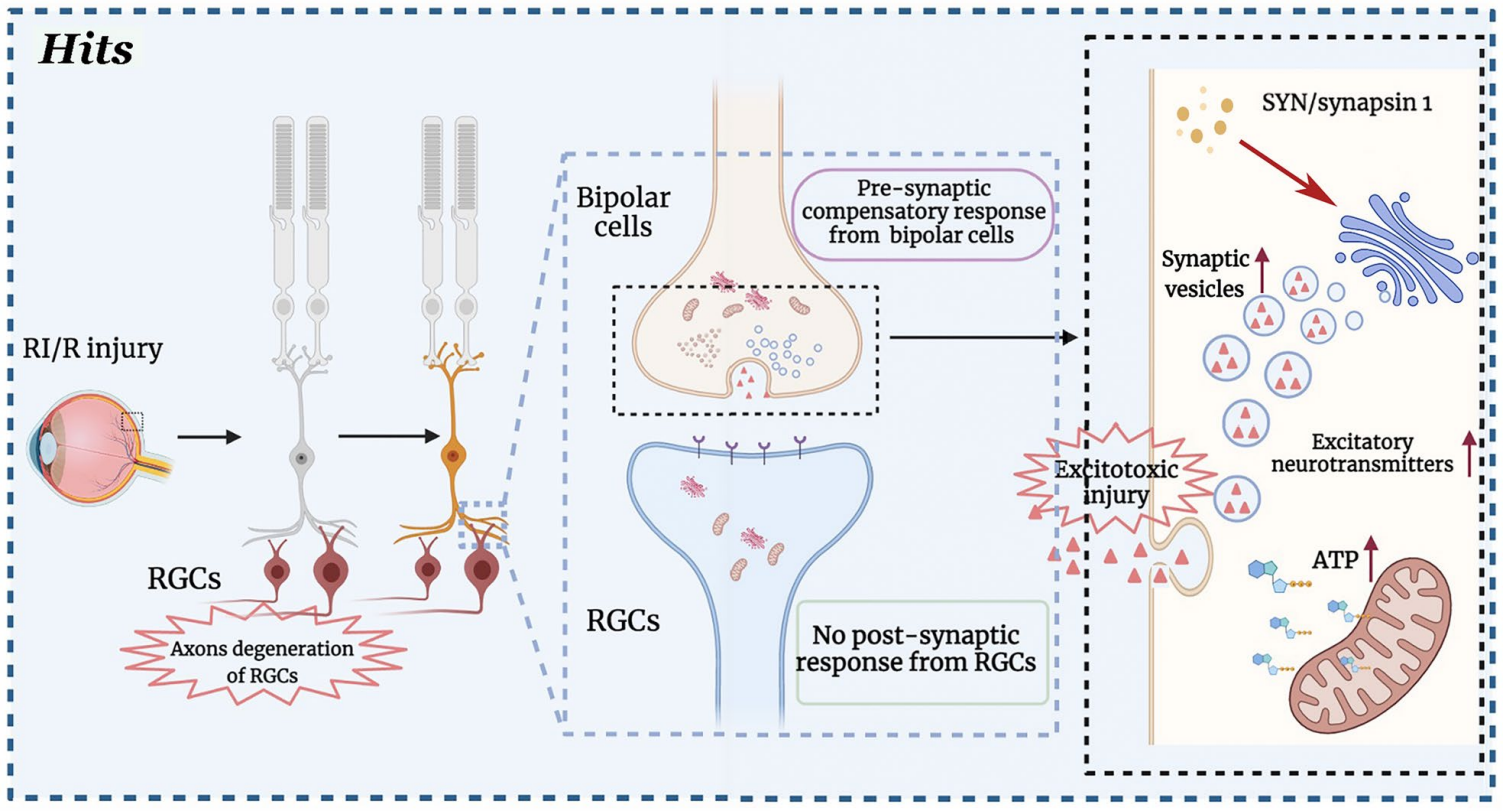
Axons degeneration of retinal ganglion cells (RGCs) following retinal ischemia/reperfusion (RI/R) triggered a synaptic compensatory response between bipolar cells and RGCs. In the normal condition, RGCs transmit the visual signals to lateral geniculate nucleus (LGN) and superior colliculus (SC) through optic nerve. This function will be weakened following the RI/R induced RGCs’ axons degeneration. In this scenario, bipolar cells strenuously synapse with RGCs to maintain, even recovery the visual function, through up-regulating synaptic vehicle proteins and enhancement of exocytosis. However, no response with post-synaptic elements occurred between bipolar cells and RGCs, which suggests that the mature synapses cannot be formed in this case. Even worse, the increased synaptic vehicle proteins and presynaptic vesicles might induce excitotoxic neurotransmitters release and excessive energy consumption. This may enhance the excitotoxic injury and increase the bipolar cells’ susceptibility to the RI/R

**Fig. 7:**
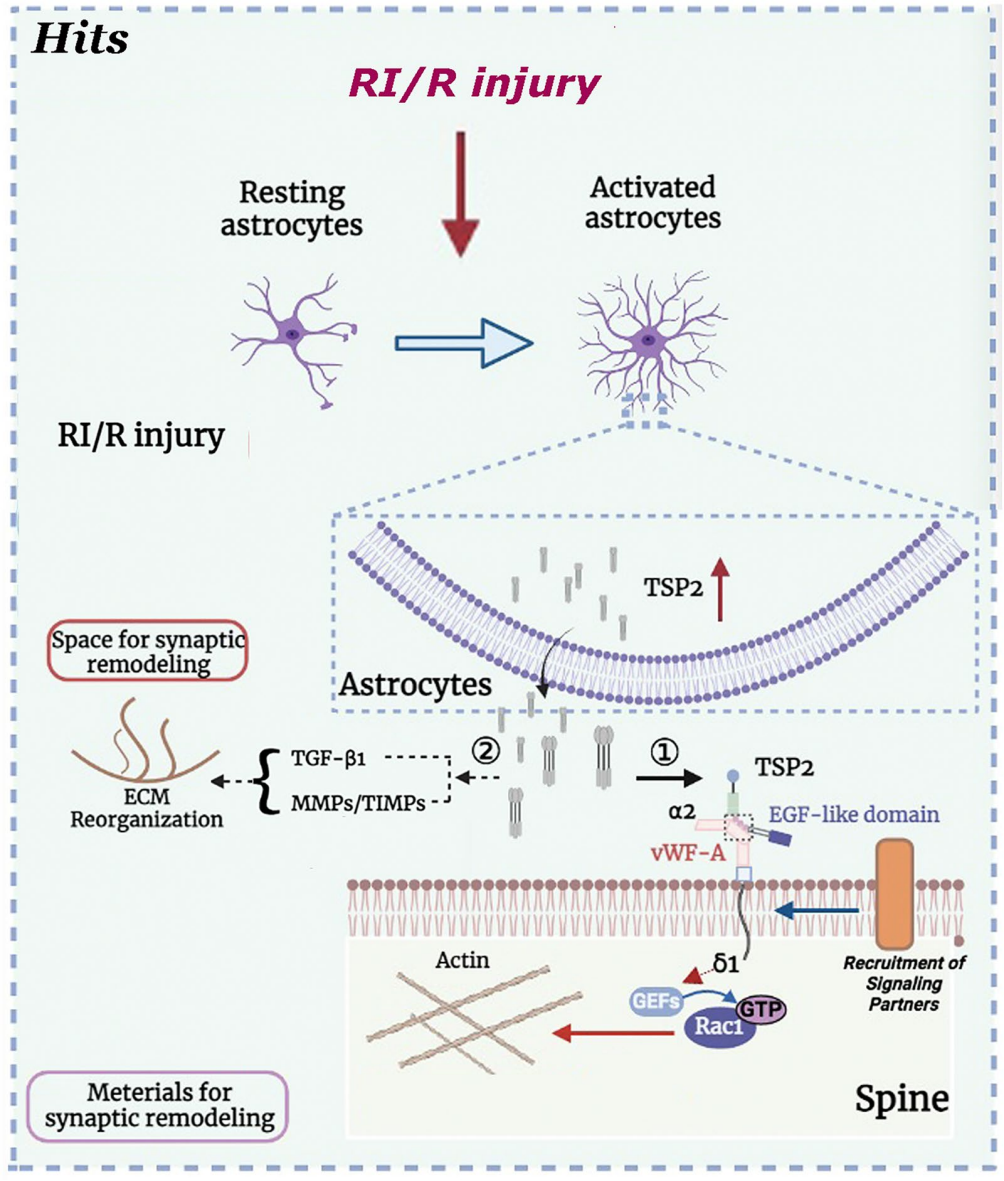
“Astrocyte-TSP2-α2δ1” pathway contributes to synaptic alteration following RI/R. RI/R switches astrocytes from “resting” to “activated”. The activated astrocytes may up-regulate and secret thrombospondin 2 (TSP 2) to bind to α2δ1, a subunit of voltage-gated calcium channels on neuronal membrane, to induce synaptic remodeling. Specifically, the EGF-like domain of TSP2 to the VWF-A domain of α2δ-1 to cause a conformational change in the molecule from closed to open. The activated α2δ-1 recruits an unidentified signal partner to combine to a “signaling complex” [56]. Then, the C terminus of α2δ-1 triggers intracellular signaling via GEFs to stimulate GTP binding to Rac1, promoting actin reorganization to facilitate spine maturation/synaptic remodeling [53]. In addition, TSP2 modulates the reorganization of the ECM by interacting with TGF-β1 and regulating the balance between MMPs and TIMPs, which provide the necessary extracellular space for synaptic remodeling [56].

We presently put forward a detailed profile of RGCs neurites and compensatory synaptic alterations at the early stages after RI/R, which may be one of the key reasons for visual dysfunction. Furthermore, the increased synaptic vehicle proteins and presynaptic vesicles might induce excitotoxic neurotransmitters release and more energy consumption in bipolar cells. This may increase bipolar cells’ susceptibility to the insults in the late stage of RI/R (Fig. 6). Further research is needed to verify these speculations and explore the potential mechanisms. In addition, we demonstrated that astrocyte and activated astrocytes-induced “TSP2-α2δ1” mediated the RGC neurites and synaptic alteration at the early stage after RI/R, which might be the potential mechanism for visual dysfunction at the early stage after RI/R injury (Fig. 7). Thus, our present study provides new evidences to retinal repair strategies for better visual function recovery on intervention times and new targets. We will further explore potential mechanisms on the role(s) of astrocytes in visual dysfunction following retinal injuries, such as functional phenotypic polarization-related, to provide new ideas for the clinical diagnosis and treatment of visual dysfunction following retinal injuries.

## Data Availability

The datasets used or analyzed during the current study are available from the corresponding author on reasonable request.

## Abbreviations

RI/R: Retinal ischemia/reperfusion
ONH: Optical nerve head
IOP: Intraocular pressure
TSP2: Thrombospondin 2
RGCs: Retinal ganglion cells
GCL: Ganglion cell layer
IPL: Inner plexiform layer
FC: Fluorocitric acid
GBP: Gabapentin
